# Investigating the Prognostic Potential of Plasma ST2 in Patients with Peripheral Artery Disease: Identification and Evaluation

**DOI:** 10.3390/proteomes12030024

**Published:** 2024-08-29

**Authors:** Ben Li, Farah Shaikh, Abdelrahman Zamzam, Rawand Abdin, Mohammad Qadura

**Affiliations:** 1 Department of Surgery, University of Toronto, Toronto, ON M5S 1A1, Canada; benx.li@mail.utoronto.ca; 2 Division of Vascular Surgery, St. Michael’s Hospital, Unity Health Toronto, University of Toronto, Toronto, ON M5B 1W8, Canada; farah.shaikh@unityhealth.to (F.S.); abdelrahman.zamzam@gmail.com (A.Z.); 3 Institute of Medical Science, University of Toronto, Toronto, ON M5S 1A1, Canada; 4 Temerty Centre for Artificial Intelligence Research and Education in Medicine (T-CAIREM), University of Toronto, Toronto, ON M5S 1A1, Canada; 5 Department of Medicine, McMaster University, Hamilton, ON L8S 4L8, Canada; rawand.abdin@medportal.ca; 6 Li Ka Shing Knowledge Institute, St. Michael’s Hospital, Unity Health Toronto, University of Toronto, Toronto, ON M5B 1W8, Canada

**Keywords:** soluble interleukin 1 receptor-like 1, biomarkers, prognosis, peripheral artery disease

## Abstract

Soluble interleukin 1 receptor-like 1 (ST2) is a circulating protein demonstrated to be associated with cardiovascular diseases; however, it has not been studied as a biomarker for peripheral artery disease (PAD). Using a prospectively recruited cohort of 476 patients (312 with PAD and 164 without PAD), we conducted a prognostic study of PAD using clinical/biomarker data. Plasma concentrations of three circulating proteins [ST2, cytokine-responsive gene-2 (CRG-2), vascular endothelial growth factor (VEGF)] were measured at baseline and the cohort was followed for 2 years. The outcome of interest was a 2-year major adverse limb event (MALE; composite of major amputation, vascular intervention, or acute limb ischemia). Using 10-fold cross-validation, a random forest model was trained using clinical characteristics and plasma ST2 levels. The primary model evaluation metric was the F1 score. Out of the three circulating proteins analyzed, ST2 was the only one that was statistically significantly higher in individuals with PAD compared to patients without PAD (mean concentration in plasma of 9.57 [SD 5.86] vs. 11.39 [SD 6.43] pg/mL, *p* < 0.001). Over a 2-year period, 28 (9%) patients with PAD experienced MALE. Our predictive model, incorporating clinical features and plasma ST2 levels, achieved an F1 score of 0.713 for forecasting 2-year MALE outcomes. Patients identified as high-risk by this model showed a significantly increased likelihood of developing MALE (HR 1.06, 95% CI 1.02–1.13, *p* = 0.003). By combining clinical characteristics and plasma ST2 levels, our proposed predictive model offers accurate risk assessment for 2-year MALE in PAD patients. This algorithm supports risk stratification in PAD, guiding clinical decisions regarding further vascular evaluation, specialist referrals, and appropriate medical or surgical interventions, thereby potentially enhancing patient outcomes.

## 1. Introduction

Over 200 million people worldwide suffer from peripheral artery disease (PAD) due to atherosclerosis of the lower extremity arteries [1,2]. Although PAD is associated with significant limb loss and mortality, it remains undertreated [3]. An important contributor to this problem is the lack of validated and standardized prognostic biomarkers that can help identify high-risk patients and guide subsequent management [3]. Therefore, there is an important need to identify and validate novel biomarkers for PAD [4].

Soluble interleukin 1 receptor-like 1 (ST2), cytokine-responsive gene-2 (CRG-2), and vascular endothelial growth factor (VEGF) are circulating protein species that have been shown to be correlated with diseases of the cardiovascular system [5,6,7]. We selected the 3 aforementioned proteins for analysis because previous studies have demonstrated strong associations between these proteoforms and cardiovascular diseases, suggesting their potential relevance to PAD [5,6,7]. Specifically, Stojkovic et al. (2020) demonstrated associations between ST2 and platelet activation in PAD patients undergoing endovascular revascularization [5], while Horrevoets and colleagues showed that CRG fragments from endothelial cells were involved in the inflammatory reaction of atherosclerotic lesions [6]. Elsewhere, Ganta et al. (2021) demonstrated that inhibition of VEGF isoforms in ischemic muscle may promote perfusion recovery in preclinical PAD [7]. While previous research has demonstrated correlations between these proteins and cardiovascular conditions, few studies have assessed their prognostic implications for PAD [5,6,7]. Furthermore, prior studies have primarily focused on individual proteins, with no previous exploration of protein biomarkers in combination with clinical characteristics for prognostic purposes.

PAD is a complex disease involving multiple metabolic pathways that are related to different proteoforms; therefore, we hypothesized that biomarkers integrated with clinical features can enhance prognostic accuracy compared to the analysis of individual proteins alone [8]. By amalgamating biomarker data with clinical features linked to PAD outcomes, there is potential to develop highly accurate and specific predictive algorithms for adverse limb events associated with PAD [9,10,11]. The goal of this study was to identify a PAD-specific biomarker and integrate clinical and circulating protein species biomarker data through predictive modeling techniques to support PAD prognosis. 

## 2. Materials and Methods

### 2.1. Ethics Approval

Approval for this study was granted by the research ethics board at Unity Health Toronto, University of Toronto, Canada on 2 August 2017 (approval code REB # 16-365). Prior to participation, informed consent was obtained from all patients, and all procedures adhered to the principles outlined in the Declaration of Helsinki [12].

### 2.2. Design

This prognostic study adhered to the Transparent Reporting of a Multivariable Prediction Model for Individual Prognosis or Diagnosis + Artificial Intelligence (TRIPOD + AI) statement [13], ensuring the transparent and comprehensive reporting of its findings.

### 2.3. Patient Recruitment

This study prospectively enrolled patients from ambulatory clinics at our institution between September 2020 and February 2022, including individuals with and without PAD. PAD was diagnosed based on an Ankle–Brachial Index (ABI) below 0.9 or a Toe Brachial Index (TBI) below 0.67, along with absent or diminished pedal pulses [14]. Patients with acute coronary syndrome, elevated troponin levels, or acute limb ischemia within the prior three months were excluded from the study.

### 2.4. Baseline Characteristics

Baseline characteristics included gender, age, dyslipidemia, hypertension, diabetes, smoking status, presence of congestive heart failure (CHF), coronary artery disease (CAD), history of stroke, and medication use including statins, acetylsalicylic acid (ASA), angiotensin II receptor blocker (ARB) or angiotensin-converting enzyme inhibitor (ACE-I), calcium channel blockers, beta-blockers, furosemide or hydrochlorothiazide, insulin, and oral antihyperglycemic agents. The definitions for cardiovascular risk factors and medications followed guidelines from the American College of Cardiology [15,16].

### 2.5. Quantification of Plasma Protein Levels

A phlebotomist collected blood samples from participants through the median cubital vein and centrifugation was used to isolate the plasma, which was then aliquoted and stored at −80 °C with no freeze–thaw cycles. On the day of analysis, the plasma was thawed to room temperature. Specifically, citrate plasma samples were used to prevent coagulation of the samples and to preserve the proteins of interest (ST2, CRG-2, and VEGF) for quantification. Using the LUMINEX assay (Bio-Techne, Minneapolis, MO, USA), in ref. [17], concentrations of three circulating proteins—ST2, CRG-2, and VEGF—were measured due to their involvement in metabolic pathways linked to atherosclerosis and cardiovascular diseases. Before analysis, the MagPix analyzer [18] was calibrated using bead kits from Fluidics Verification and Calibration (Luminex Corp; Austin, TX, USA) [19]. To ensure consistency, all tests were performed on the same day to minimize variability between assays. The inter- and intra-assay coefficients of variability for the samples were less than 10%. Luminex xPonent software version 4.3 [20] was used to evaluate a minimum of 50 beads per protein. The lower limit of detection (LLOD) and lower limit of quantification (LLOQ) of the Luminex multiplex assays were in the 0.1–1 pg/mL range for the proteins of interest [21]. Given that the mean plasma concentrations of the proteins of interest (ST2, CRG-2, and VEGF) ranged from 9.57 to 42.5 pg/mL in our cohort, all samples were measurable.

### 2.6. Follow-Up and Outcomes

Follow-up clinic visits were conducted at 12- and 24-months post-recruitment. The primary outcome of interest was major adverse limb events (MALE) occurring within 2 years of recruitment. MALE was defined as the need for major amputation of the lower extremity above the ankle, vascular intervention (either open or endovascular revascularization of the lower extremity arteries), or acute limb ischemia (sudden decrease in limb perfusion occurring within 14 days due to arterial thrombosis or embolism). Initial analysis revealed that all adverse limb events occurred exclusively in PAD patients. Therefore, prognostic models were developed solely within the PAD cohort.

### 2.7. Model Development and Evaluation

Random forest was chosen as the predictive algorithm, which is an ensemble model that works through several decision trees [22]. Decision trees can classify samples into branch-like segments and leverage data from multiple covariates to develop prediction algorithms for an outcome of interest [23]. Random forest can leverage complex datasets effectively because of its non-parametric nature [23]. This algorithm was chosen because it is well described in literature and performs well for predicting human health outcomes [24,25,26].

The dataset was partitioned randomly into 70% for training and 30% for testing purposes. Utilizing the random forest algorithm, which underwent training with 10-fold cross-validation, our aim was to predict 2-year MALE. The input features comprised clinical characteristics such as gender, age, diabetes, dyslipidemia, hypertension, smoking status, CAD, CHF, previous stroke, and medication use (including statins, ASA, ARB or ACE-I, beta-blockers, calcium channel blockers, furosemide or hydrochlorothiazide, insulin, and oral antihyperglycemic agents), alongside plasma ST2 levels. Post-training, the models were evaluated on unseen test set data. Variable importance scores (gain) were calculated to identify the most influential predictive factors, gauging their relative impact on prediction outcomes [27].

### 2.8. Statistical Analysis

We summarized the baseline characteristics using means and standard deviations (SDs) for continuous variables or numbers and proportions for categorical variables. Group differences were assessed using independent t-tests for continuous variables and chi-square tests for categorical variables. Protein levels were compared between PAD and non-PAD patients using independent t-tests. Proteins that showed differential expression in patients with versus without PAD were further utilized in model development. Two-year event rates were compared between patients with vs. without PAD using chi-square tests. Hazard ratios were calculated to determine the association between ST2 and adverse limb events controlling for all baseline characteristics. The F1 score was the primary model evaluation metric, which measures the harmonic mean of the precision and recall values by which ST2 levels predicted adverse limb events [28]. The F1 score, defined by the formula 2 × (precision × recall)/(precision + recall), ranges from 0 to 1, where 0 indicates minimal precision and/or recall, and 1 signifies optimal precision and recall [28]. Using our prognostic model, patients were stratified into low- or high-risk categories for developing 2-year MALE based on the Youden Index, which maximizes the model’s performance (sensitivity and specificity) through receiver operating characteristic (ROC) curve analysis [29]. To assess freedom from MALE over 2 years between low- and high-risk groups, Kaplan–Meier curves were plotted and compared using Cox proportional hazards analysis, adjusting for all baseline characteristics. This stratified analysis aimed to elucidate the clinical relevance of risk predictions derived from the prognostic model, offering insights into how the trajectories of low-risk versus high-risk patients differ in terms of MALE risk over a 2-year period. Statistical significance was set at a two-tailed *p*-value < 0.05. All statistical analyses were conducted using SPSS software version 23 (SPSS Inc., Chicago, IL, USA) [30].

## 3. Results

### 3.1. Patients

In total, 476 patients participated in this study, with 312 having PAD and 164 without PAD. Those with PAD were older (mean age 71 [SD 10] vs. 65 [SD 12] years; *p* < 0.001) and a higher percentage had CAD (38% vs. 21%; *p* < 0.001), diabetes (42% vs. 21%; *p* < 0.001), dyslipidemia (84% vs. 61%; *p* < 0.001), hypertension (82% vs. 59%; *p* < 0.001), and a history of stroke (16% vs. 8%; *p* = 0.011). They were also more likely to be current/past smokers (80% vs. 64%; *p* = 0.002). Additionally, individuals with PAD were more frequently prescribed risk-reduction medications such as ACE-I/ARB (66% vs. 45%, *p* = 0.001), statins (73% vs. 57%, *p* < 0.001), ASA (80% vs. 60%, *p* < 0.001), and beta blockers (41% vs. 30%, *p* = 0.001) (Table 1).

### 3.2. Plasma Protein Concentrations

Of the three proteins tested, ST2 was the only protein that was statistically significantly higher in individuals with PAD compared to patients without PAD: mean concentration in plasma of 11.39 [SD 6.43] vs. 9.57 [SD 5.86] pg/mL; *p* < 0.001 (Table 2). ST2 was therefore included in further analyses.

### 3.3. Adverse Limb Events

All adverse limb events observed during the 2-year follow-up period were exclusively found in patients with PAD: MALE (n = 28, 9%), major amputation (n = 17, 5%), and vascular intervention (n = 19, 6%). No patients developed acute limb ischemia (Table 3). There were significant associations between ST2 and adverse events over 2 years of follow-up, including MALE (HR 1.06 [95% CI 1.02–1.13], *p* = 0.005) and need for vascular intervention (HR 1.07 [95% CI 1.01–1.12], *p* = 0.003) (Table 4). Of note, Table 1 and Table 3 are similar to our previous publication because we used a similar patient cohort to investigate PAD biomarkers [31]. While our previous paper identified IL-7 as a myokine biomarker for PAD, the current study is innovative in that we discovered a novel PAD biomarker, ST2, which has a unique biological relationship with PAD through its involvement in various mechanistic pathways of atherosclerosis, thrombosis, and plaque vulnerability. Therefore, the current study adds to our previous work by unveiling a new PAD biomarker that can further guide our understanding of PAD pathophysiology and prognosis.

### 3.4. Model Performance

**Figure 1:**
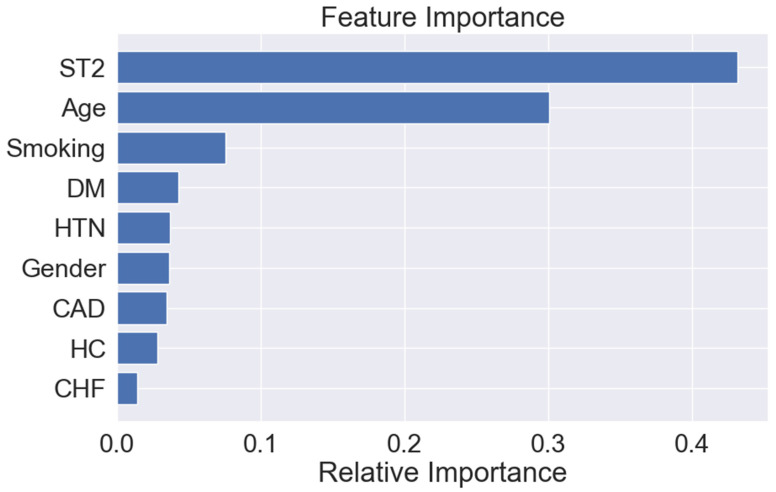
Variable importance scores (gain) for the random forest model input features for prognosis of 2-year major adverse limb events in individuals with peripheral artery disease. Abbreviations: soluble interleukin 1 receptor-like 1 (ST2), congestive heart failure (CHF), coronary artery disease (CAD), diabetes mellitus (DM), hypercholesterolemia (HC), hypertension (HTN).

Using a combination of clinical features and plasma ST2 levels, the random forest model achieved an F1 score of 0.713 for predicting 2-year MALE. The most important predictive features for the model were (1) ST2, (2) age, (3) current/past smoking, (4) diabetes, (5) hypertension, (6) gender, (7) CAD, (8) dyslipidemia, and (9) CHF (Figure 1). Of note, ST2 was the most important predictive feature for 2-year MALE when evaluated alongside patient age, gender, and comorbidities as input features.

### 3.5. Risk Stratification Using Model

**Figure 2:**
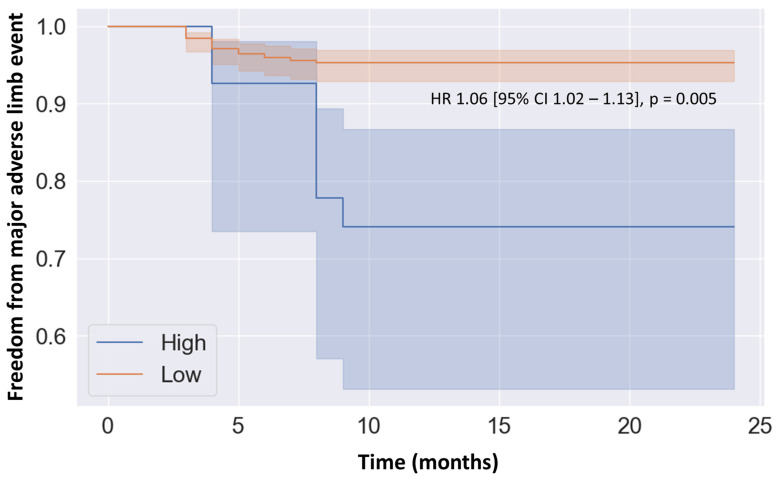
Kaplan–Meier analysis of the freedom from major adverse limb events among patients categorized as low versus high risk by the random forest model. Classification into these risk groups was based on a threshold of 0.60 derived from the Youden Index, optimizing the prediction model’s sensitivity and specificity through receiver operating characteristic curve analysis. Cox proportional hazards analysis adjusted for sex, age, dyslipidemia, hypertension, diabetes, smoking history, coronary artery disease, congestive heart failure, previous stroke, use of statins, acetylsalicylic acid, angiotensin II receptor blocker or angiotensin-converting enzyme inhibitor, calcium channel blocker, beta blocker, furosemide or hydrochlorothiazide, oral antihyperglycemic agent, and insulin. Abbreviations: CI (confidence interval), HR (hazard ratio).

Youden’s Index calculation identified 0.60 as the optimal threshold for predicting 2-year MALE using our model. This threshold was employed to categorize our cohort into high and low-risk groups for adverse limb events. Over the 2-year follow-up period, patients classified as high-risk exhibited reduced freedom from MALE compared to those classified as low-risk (HR 1.06, 95% CI 1.02–1.13, *p* = 0.005) (Figure 2).

## 4. Discussion

### 4.1. Summary of Findings

In this study, we identified ST2 as a specific proteoform biomarker for PAD and developed a robust prognostic model combining clinical characteristics and plasma ST2 levels to accurately predict PAD prognosis. Several key findings emerged from our analysis. Firstly, among the three circulating protein species studied, ST2 was the sole proteoform found to be elevated in individuals with PAD compared to those without PAD. Although this study investigated multiple proteins as potential PAD biomarkers, only ST2 emerged as the singular protein that was significantly correlated with PAD-related adverse limb events. Therefore, ST2 was identified as the most robust PAD-specific prognostic biomarker. Secondly, our predictive model, integrating clinical features and plasma ST2 levels, demonstrated strong performance in forecasting PAD prognosis. Feature importance analysis underscored ST2 levels as the most significant predictor, highlighting the pivotal role of proteoform biomarkers in predicting outcomes in PAD. Although additional research is needed to confirm our findings, our study demonstrates that ST2 can be considered a PAD-specific biomarker, as it was the only protein that was significantly elevated in patients with PAD and predicted the development of adverse limb events over a 2-year follow-up period. Given the prognostic relevance of ST2, further research is warranted to explore its biological relationship with PAD development and progression, aiming to enhance our understanding of proteome complexity and inform targeted therapeutic strategies. Thirdly, utilizing our prognostic model, we stratified patients into low- and high-risk categories for adverse events. Kaplan–Meier analysis illustrated that patients identified as high-risk by our model were more susceptible to developing 2-year MALE compared to those classified as low-risk. This underscores the clinical utility of our model in aiding clinicians to predict the future risk trajectory of PAD patients concerning adverse limb events.

### 4.2. Comparison to Existing Literature

Ross and colleagues (2019) developed a prognostic model for predicting major adverse cardiac and cerebrovascular events in PAD patients using electronic health records data [32]. However, their model did not incorporate biomarker-based input features, despite the demonstrated impact of proteoform biomarkers on PAD prognosis in our study and others [5,6,7]. Our research addressed this limitation by integrating protein species biomarker data into our prediction models. We achieved strong performance in predicting 2-year MALE among PAD patients, with an F1 score of 0.713. Thus, our study underscores the importance of utilizing proteoform biomarker data to enhance prognostic models, potentially improving their predictive accuracy compared to models based solely on clinical features.

### 4.3. Explanation of Findings

There are several potential explanations for our findings. First, ST2 was found to be significantly elevated in patients with PAD in this study and was an important predictor of PAD prognosis. Discovered in 1989, the ST2 gene is found on chromosome 2q12 as part of the wider interleukin 1 (IL-1) gene cluster [33]. ST2 is the IL-33 receptor and facilitates downregulation of hypertrophy and fibrosis in tissues that are mechanically strained [34]. ST2 has been demonstrated to be associated with the pathogenesis of atherosclerosis and thrombosis in previous studies [35,36]. Specifically, in monocytes, ST2 proteoforms induce tissue factor expression and the release of prothrombotic vesicles [37]. Consequently, increased levels of IL-33/ST2 are associated with an increased risk of in-stent restenosis and poor outcomes in patients with CAD [38,39]. Similarly, in patients with carotid artery stenosis, ST2 levels have been found to be elevated and correlated with atherosclerotic plaque vulnerability [40]. ST2 has been shown to be a potential biomarker in individuals with heart failure and myocardial infarction as it is correlated with adverse cardiovascular events and death [41,42]. Taken together, these findings explain the potential mechanism by which ST2 is involved in PAD development. In comparison to our previously published work [43,44,45,46,47], we identified ST2 as a novel prognostic biomarker for PAD that has excellent predictive performance for adverse limb events in combination with clinical features. Importantly, ST2 has a strong biological relationship to PAD through its involvement in various mechanistic pathways of atherosclerosis, thrombosis, and plaque vulnerability. Therefore, there is potential to combine ST2 with our previously identified PAD biomarkers to form a comprehensive panel of proteins to support PAD management. This may form the basis for a larger future validation study. Secondly, our analysis demonstrated a high incidence of adverse limb events in PAD patients, with close to 10% of the cohort experiencing MALE over 2 years. This highlights the need for new strategies to reduce complications in this patient population, emphasizing the value of developing more effective prognostic tools. Third, our prognostic model achieved excellent performance for several potential reasons. Contrary to traditional statistics such as logistic regression, which assumes that there is a linear correlation between the covariates and the logit of the dependent variable, advanced modeling methods are not limited by the linearity assumption and can therefore better model non-linear relationships [48,49]. This is valuable in proteoform biomarker-based prognostic models, where different protein species may be involved in various biological pathways that may interact in complex ways because of proteome complexity to contribute to a disease condition [50]. Our random forest algorithm likely achieved good performance because it is an ensemble model that combines several decision trees [51]. This approach minimizes overfitting, reduces variance, and effectively handles complex datasets [51]. Our study highlights the advantages of developing a prognostic model that includes proteoform biomarker information, which may enhance model performance in comparison to solely using clinical information. Because PAD is a complex condition that involves multiple biological pathways, previous papers have shown the value of an integrated approach to support the prognosis of PAD [52]. This investigation affirms that by applying advanced predictive modeling methods to assess clinical information in conjunction with biomarker data, accurate PAD prognostic tools can be generated.

### 4.4. Implications

Our predictive models offer practical implications for guiding clinical decision-making across various scenarios. First, our algorithm can help screen individuals for asymptomatic PAD, which is especially valuable in family practice settings. General practitioners use the predictive model during their clinical assessments to understand a patient’s PAD risk [53]. Patients who screen positive for being at elevated risk of PAD-associated adverse outcomes may be sent for additional vascular evaluation, including arterial duplex ultrasound, to confirm a PAD diagnosis [54]. Patients identified as low-risk may receive ongoing treatment from their family physician with a focus on risk factor optimization such as lifestyle modifications and prescription of statins and ASA [55]. Patients predicted to be at elevated risk for MALE should be referred to a vascular surgeon for evaluation and treatment [56]. Vascular surgeons can integrate our model with their clinical judgment to identify individuals at heightened risk of adverse limb outcomes, potentially benefiting from the following: (1) additional imaging studies to evaluate vascular anatomy and disease severity [57], (2) low-dose rivaroxaban therapy [58], or (3) surgical interventions for limb salvage [59,60]. Our predictive model has the potential to enhance care for individuals with PAD across both generalist and specialist healthcare settings. It can facilitate PAD screening, risk stratification, and early identification of patients at increased risk for adverse limb outcomes. This approach may reduce unnecessary specialist referrals, improve PAD management outcomes, and decrease healthcare expenditures [61].

### 4.5. Limitations

Our study has several limitations. Firstly, recruitment occurred at a single center, and validation in other settings may help determine model generalizability. Notably, the non-PAD group had a smaller sample size compared to the PAD group because the focus of this study was to identify a prognostic biomarker for PAD patients. However, it may be prudent for future studies to include a larger non-PAD cohort as a control group. Secondly, the outcomes in our study were recorded based on a 2-year follow-up period. Additional follow-up may improve our understanding of the predictive ability of our model given that PAD is a chronic condition. Given that the goal of this study was to identify prognostic biomarkers for PAD, the correlation between ST2 and other cardiovascular diseases was not investigated. Thirdly, laboratory results such as lipid levels, creatinine, liver function tests, glucose, and coagulation parameters were not measured in this study. Future models incorporating these variables may further improve predictive performance. Fourthly, the protein species biomarkers evaluated in this paper are used predominantly in research environments. Additional work is required to demonstrate the clinical feasibility of including these proteoform biomarkers in the routine care of patients with PAD.

## 5. Conclusions

We identified ST2 as a protein species biomarker for PAD and used plasma concentrations of ST2 in combination with clinical characteristics to build an algorithm that accurately predicts PAD prognosis. Our model holds promise for PAD risk stratification, supporting prompt identification and targeted treatment of PAD. Specifically, the model can identify high-risk patients who may be referred for additional vascular work-up and may benefit from more aggressive medical and/or surgical management. Additionally, our paper highlights the need for translational research assessing the biological relationship between ST2 and PAD progression, offering potential advancements in our understanding of the underlying pathogenesis of PAD and informing targeted therapeutic strategies.

## Figures and Tables

**Table 1:** Baseline characteristics.

	PAD (n = 312)	Non-PAD (n = 164)	*p*
Age, mean (SD)	71 (10)	65 (12)	<0.001
Female sex	109 (35)	67 (41)	0.204
Hypertension	257 (82)	96 (59)	<0.001
Diabetes	131 (42)	34 (21)	<0.001
Dyslipidemia	263 (84)	100 (61)	<0.001
Current smoking	78 (25)	35 (21)	0.002
Past smoking	171 (55)	71 (43)	0.001
Coronary artery disease	118 (38)	34 (21)	<0.001
Congestive heart failure	11 (4)	4 (2)	0.519
Previous stroke	51 (16)	13 (8)	0.011
Statin	229 (73)	93 (57)	<0.001
Acetylsalicylic acid	251 (80)	99 (60)	<0.001
Beta blocker	134 (41)	50 (30)	0.001
ACE-I/ARB	216 (66)	74 (45)	0.001
Calcium channel blocker	82 (25)	34 (21)	0.079
Hydrochlorothiazide or furosemide	41 (13)	17 (10)	0.190
Insulin	22 (7)	6 (4)	0.255
Oral antihyperglycemic agent	24 (8)	8 (5)	0.201

Values are presented as number (percentage) unless specified otherwise. ARB (angiotensin II receptor blocker), ACE-I (angiotensin-converting enzyme inhibitor), PAD (peripheral artery disease), SD (standard deviation).

**Table 2:** Protein plasma concentrations in individuals with vs. without peripheral artery disease.

	Non-PAD (n = 164)		PAD (n = 312)		
	Mean	Standard Deviation	Mean	Standard Deviation	*p*
ST2	9.57	5.86	11.39	6.43	<0.001
CRG-2	38.56	28.56	42.5	30.46	0.085
VEGF	16.61	12.24	20.81	26.96	0.096

Protein concentrations reported in pg/mL. Abbreviations: soluble interleukin 1 receptor-like 1 (ST2), cytokine-responsive gene-2 (CRG-2), vascular endothelial growth factor (VEGF).

**Table 3:** Two-year adverse limb events.

	PAD (n = 312)	Non-PAD (n = 164)	*p*
Major adverse limb event	28 (9)	0 (0)	0.001
Major amputation	17 (5)	0 (0)	0.002
Vascular intervention	19 (6)	0 (0)	0.001
Acute limb ischemia	0 (0)	0 (0)	N/A

Values are presented as number (percentage) unless specified otherwise. PAD (peripheral artery disease).

**Table 4:** Adjusted hazard ratios for associations between soluble interleukin 1 receptor-like 1 (ST2) and 2-year major adverse limb events in patients with peripheral artery disease.

	Hazard Ratio [95% CI] *	*p*-Value
Major adverse limb event	1.06 [1.02–1.13]	0.005
Vascular intervention	1.07 [1.01–1.12]	0.003
Major amputation	1.00 [0.99–1.11]	0.084

* Adjusted for age, sex, hypertension, dyslipidemia, diabetes, past/current smoking, congestive heart failure, coronary artery disease, previous stroke, acetylsalicylic acid, statin, angiotensin-converting enzyme inhibitor or angiotensin II receptor blocker, beta blocker, calcium channel blocker, hydrochlorothiazide or furosemide, oral antihyperglycemic agent, and insulin.

## Data Availability

The original contributions presented in the study are included in the article, further inquiries can be directed to the corresponding author.
